# Functional analysis of *PagNAC045* transcription factor that improves salt and ABA tolerance in transgenic tobacco

**DOI:** 10.1186/s12870-022-03623-8

**Published:** 2022-05-25

**Authors:** Xuemei Zhang, Zihan Cheng, Gaofeng Fan, Wenjing Yao, Wei Li, Sixue Chen, Tingbo Jiang

**Affiliations:** 1 State Key Laboratory of Tree Genetics and Breeding, Northeast Forestry University, 51 Hexing Road, Harbin, 150040 China; 2 Department of Biology, Genetics Institute, University of Florida (UF), Gainesville, FL 32611 USA; 3 Co-Innovation Center for Sustainable Forestry in Southern China/Bamboo Research Institute, Nanjing Forestry University, 159 Longpan Road, Nanjing, 210037 China; 4 Plant Molecular and Cellular Biology Program, UF, Gainesville, FL 32610 USA; 5 Proteomics and Mass Spectrometry, Interdisciplinary Center for Biotechnology Research, UF, Gainesville, FL 32610 USA

**Keywords:** *Populus alba×P. glandulosa*, *PagNAC045*, Transcription factor, Tobacco, Salt stress, ABA treatment

## Abstract

**Background:**

Salt stress causes inhibition of plant growth and development, and always leads to an increasing threat to plant agriculture. Transcription factors regulate the expression of various genes for stress response and adaptation. It’s crucial to reveal the regulatory mechanisms of transcription factors in the response to salt stress.

**Results:**

A salt-inducible NAC transcription factor gene *PagNAC045* was isolated from *Populus alba×P. glandulosa*. The *Pag*NAC045 had a high sequence similarity with NAC045 (Potri.007G099400.1) in *P. trichocarpa*, and they both contained the same conserved motifs 1 and 2, which constitute the highly conserved NAM domain at the N-terminus. Protein-protein interaction (PPI) prediction showed that *Pag*NAC045 potentially interacts with many proteins involved in plant hormone signaling, DNA-binding and transcriptional regulation. The results of subcellular localization and transient expression in tobacco leaves confirmed the nuclear localization of *Pag*NAC045. Yeast two-hybrid revealed that *Pag*NAC045 protein exhibits transcriptional activation property and the activation domain located in its C-terminus. In addition, the 1063 bp promoter of *PagNAC045* was able to drive *GUS* gene expression in the leaves and roots. In poplar leaves and roots, *PagNAC045* expression increased significantly by salt and ABA treatments. Tobacco seedlings overexpressing *PagNAC045* exhibited enhanced tolerance to NaCl and ABA compared to the wild-type (WT). Yeast one-hybrid assay demonstrated that a bHLH104-like transcription factor can bind to the promoter sequence of *PagNAC045*.

**Conclusion:**

The *PagNAC045* functions as positive regulator in plant responses to NaCl and ABA-mediated stresses.

**Supplementary Information:**

The online version contains supplementary material available at 10.1186/s12870-022-03623-8.

## Background

Plants often encounter different abiotic stresses, such as extreme temperatures, ultraviolet radiation, water deficits, oxidative stress, heavy metal toxicity, drought, and salinity [1]. These stresses pose a severe threat to plant growth and development, crop productivity and ecosystem balance worldwide [2]. Among them, salt stress is considered as the most significant environmental challenge, causing osmotic and toxic effect, oxidative damage, physiological water deficit, nutritional imbalance, and metabolic perturbation during plant growth and development [3–7]. In addition, soil salinization has been a primary global problem for a long time because of natural and human-induced actions [8]. However, the knowledge about the mechanisms of salt response in woody plants is scarce. *P. alba×P. glandulosa* (84 K) is a male *P. alba×P. tremula var. glandulosa* interspecific hybrid, and has been a representative model woody plant in plant stress biology research [9–11]. It has the advantages of easy rooting, short seedling period and strong tolerance to abiotic stresses [12], including drought [13], salt [14], cadmium [15], and light stress [16]. Additionally, 84 K poplar is widely used for timber, firewood, and pulp production [11].

Abscisic acid (ABA) is one of important phytohormones in plant stress response (e.g., to drought) [17]. Its induced biosynthesis appears to be one of the fastest responses to abiotic stresses [18]. Both ABA-dependent and ABA-independent pathways are involved in the regulation of plant stress response and osmotic stress-responsive gene expression [19, 20]. During the response process, ABA acts as a critical regulator in plant cells to cause significant physiological changes [21], e.g., activating ABA-induced gene expression and stomatal closure to reduce water loss [22]. Salt stress triggers osmotic stress signaling and ABA pathways [23]. However, the relationship between ABA and salt response in plants still needs to be uncovered.

Transcription factors (TFs) are key regulatory proteins that play fundamental roles in various biological processes and regulate different metabolic, developmental and stress response pathways by binding to specific *cis*-elements to control gene expression [24]. To date, 58 TF families were recorded including AP2, ERF, HD-ZIP, HSF, and NAC on PlantTFDB (http://planttfdb.gao-lab.org/index.php). Here we focus on NAC (NAM, ATAF1,ATAF2, CUC2) TFs [25], which contain a highly conserved N-terminal NAC domain for DNA-binding and dimerization capability, and a highly variable C-terminal regulatory domain for transcriptional activation or repression function [26, 27]. According to the PlantTFDB, 138, 228, 280, 155, 289, 189, and 145 NACs have been identified in *Arabidopsis thaliana*, *Nicotiana benthamiana*, *N. tabacum*, *P. euphratica*, *P. trichocarpa*, *Zea mays*, and *Citrus sinensis*, respectively. NACs have been reported to be involved in various plant processes, such as cell division [28], wood formation [29], and plant senescence [30]. Besides, NACs also play a crucial role in response to different environmental stresses [31–33]. For example, a NAC TF *SlJUB1* gene induced by various abiotic stresses can control the expression of *SlDREB1*, *SlDRED2*, and *SlDELLA* to enhance tomato drought tolerance [34]. Overexpression of *NAC13* can improve the salt stress tolerance in both transgenic poplar [35] and tobacco plants [36]. Overexpression of an *OsNAC066* in rice improved drought and oxidative stress tolerance and increased ABA sensitivity [37]. In addition, 289 NAC TFs were retrieved and quantified based on RNA-seq datasets in our previous studies [38–40]. Based on log2 fold change (FC) ≥1 and false discovery rate (FDR) ≤0.05, 37 genes were significantly up-regulated under salt stress [40] and these genes could be vertically clustered into three groups. The *PagNAC045* TF displayed increased expression under salt stress and was classified into the same group with *PagNAC13* (Potri.001G404100.1) [35] and *PagNAC036* (Potri.011G123300.1) [40].

In this study, we focused on the *PagNAC045* TF gene from the 84 K poplar. The expression of *PagNAC045* was significantly induced by salt stress [39, 40]. To better understand the characteristics of *PagNAC045*, we cloned the gene from the 84 K poplar leaves based on the sequence of a homolog *NAC045* from *P. trichocarpa.* Next, we amplified the promoter sequence of the *PagNAC045* to identify the upstream regulatory elements using the yeast one-hybrid system. Additionally, to reveal the function of *PagNAC045* in response to salinity and ABA, we obtained *PagNAC045-*overexpressing transgenic tobacco lines by *Agrobacterium*-mediated transformation, and treated them with 200 mM NaCl and 50 μM ABA. Physiological parameters and the expression of downstream stress-related genes were analyzed. These results provide insight into the function of *PagNAC045* in plant response to salt stress.

## Results

### Bioinformatic characterization of *PagNAC045* transcription factor

**Fig. 1:**
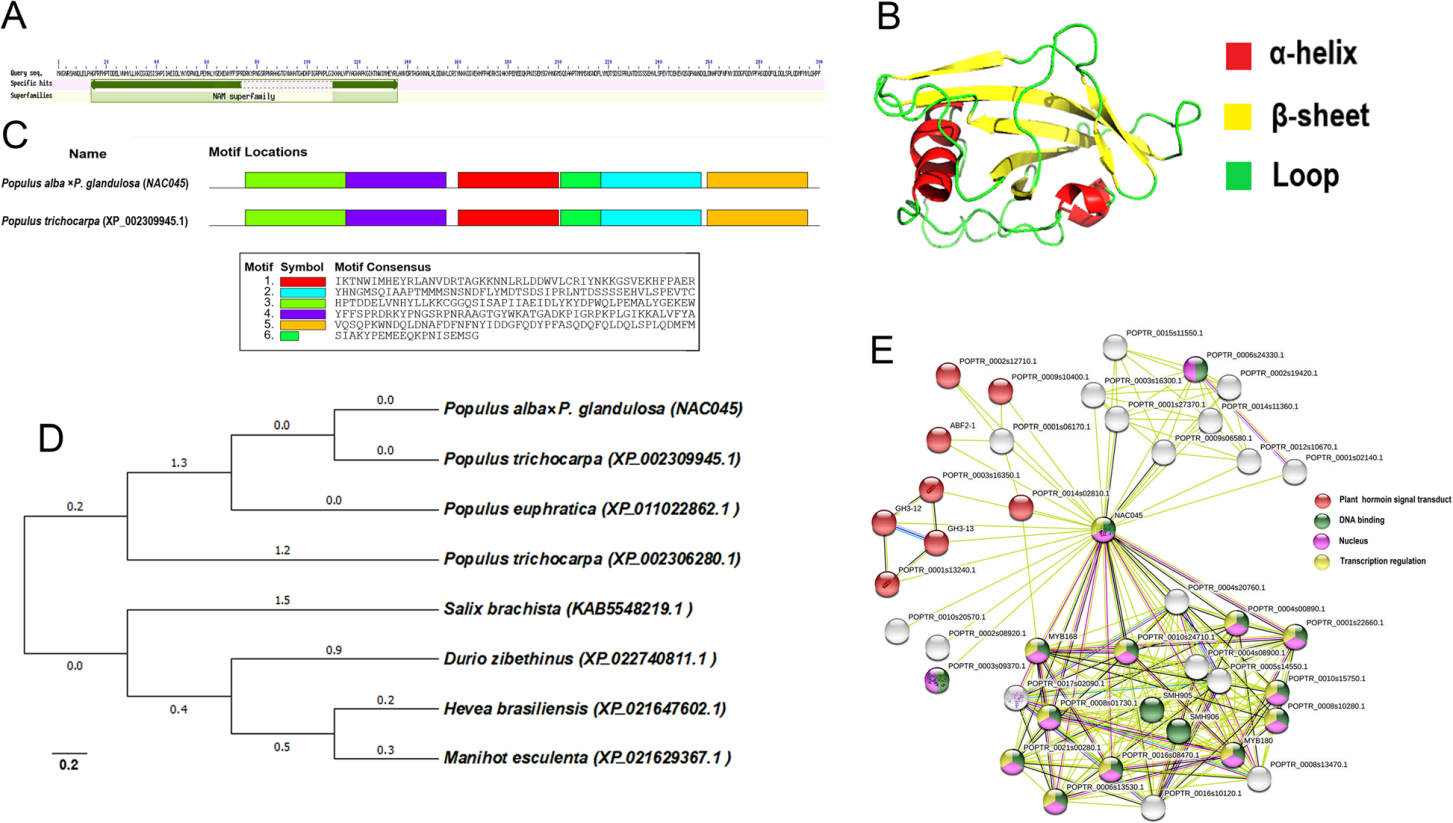
Bioinformatic analysis of the poplar *PagNAC045* gene and protein. **A** Structure of the *Pag*NAC045 protein. **B** Conserved motifs composition of the *Pag*NAC045 and homologous proteins in *Populus trichocarpa*. **C** *Pag*NAC045 protein structure prediction. **D** Phylogenetic tree analysis of the *Pag*NAC045 by MEGA7 with the Neighbor Joining method. **E** Functional protein association networks of the *Pag*NAC045 by STRING

The *PagNAC045* gene of 84 K poplar is 915 bp in length to encode a protein of 305 amino acids (aa), which contains a highly conserved NAM domain at its N-terminus (Fig. 1A). The protein consists of 16.78% alpha helix, 11.18% extended strand, 3.62% beta turn and 68.42% random coil (Fig. 1B). According to NCBI protein blast result, the *Pag*NAC045 amino acid sequence had 97.04% similarity to NAC045 (XP_002309945.1, Potri.007G099400.1) of *P. trichocarpa*, and they share six highly conserved motifs. Motifs 1 and 2 consist of the conserved NAM domain at N-terminus (Fig. 1C). Seven highly homologous proteins were found from the NCBI database. They include *P. trichocarpa* (XP_002309945.1), *P. euphratica* (XP_011022862.1), *Salix brachista* (KAB5548219.1), *P. trichocarpa* (XP_002306280.1), *Hevea brasiliensis*, *Manihot esculenta* and *Durio zibethinus*, sharing 97.04, 96.38, 90.46, 88.52, 80.13, 78.98 and 75.08% sequence similarity with the *Pag*NAC045, respectively (Fig. 1D). Based on the results of STRING analysis, 40 proteins were predicted to form a network (Fig. 1E and Supplementary Table S1), with an average node degree of 10.8, a local clustering coefficient of 0.845, and a PPI enrichment *p*-value < 1.0e-16. Among these proteins, some are involved in the process of plant hormone signal transduction (red ball) including bZIP TF 6 family protein POPTR_0002s12710.1 and POPTR_0014s02810.1, ABA-insensitive 5-like protein 7 POPTR_0009s10400.1, ABA responsive elements-binding protein 2 ABF2-1, jasmonate (JA) zim domain-containing protein POPTR_0003s16350.1, JA-amido synthetase JAR1 GH3-12 and GH3-1, and JA zim domain-containing protein POPTR_0001s13240.1. Besides, some proteins play crucial roles in DNA binding (green ball), in nucleus (purple ball), and functioning as transcription regulators (yellow ball). Additionally, 10 proteins connected with black lines were predicted to be co-expressed with the *Pag*NAC045. These include zinc finger CCCH domain-containing protein 30 POPTR_0001s27370.1 and POPTR_0009s06580.1, Tau class glutathione transferase GSTU45 POPTR_0016s10120.1, WRKY TF 42 family protein POPTR_0021s00280.1, syringolide-induced protein 1-3-1B POPTR_0008s01730.1, probable WRKY TF 48 POPTR_0010s15750.1 and its isoform POPTR_0008s10280.1, WRKY TF 6 family protein POPTR_0004s00890.1, TF salt-related MYB1 (srm1) POPTR_0010s24710.1 and POPTR_0001s22660.1.

### Subcellular localization of *Pag*NAC045 protein in tobacco

**Fig. 2:**
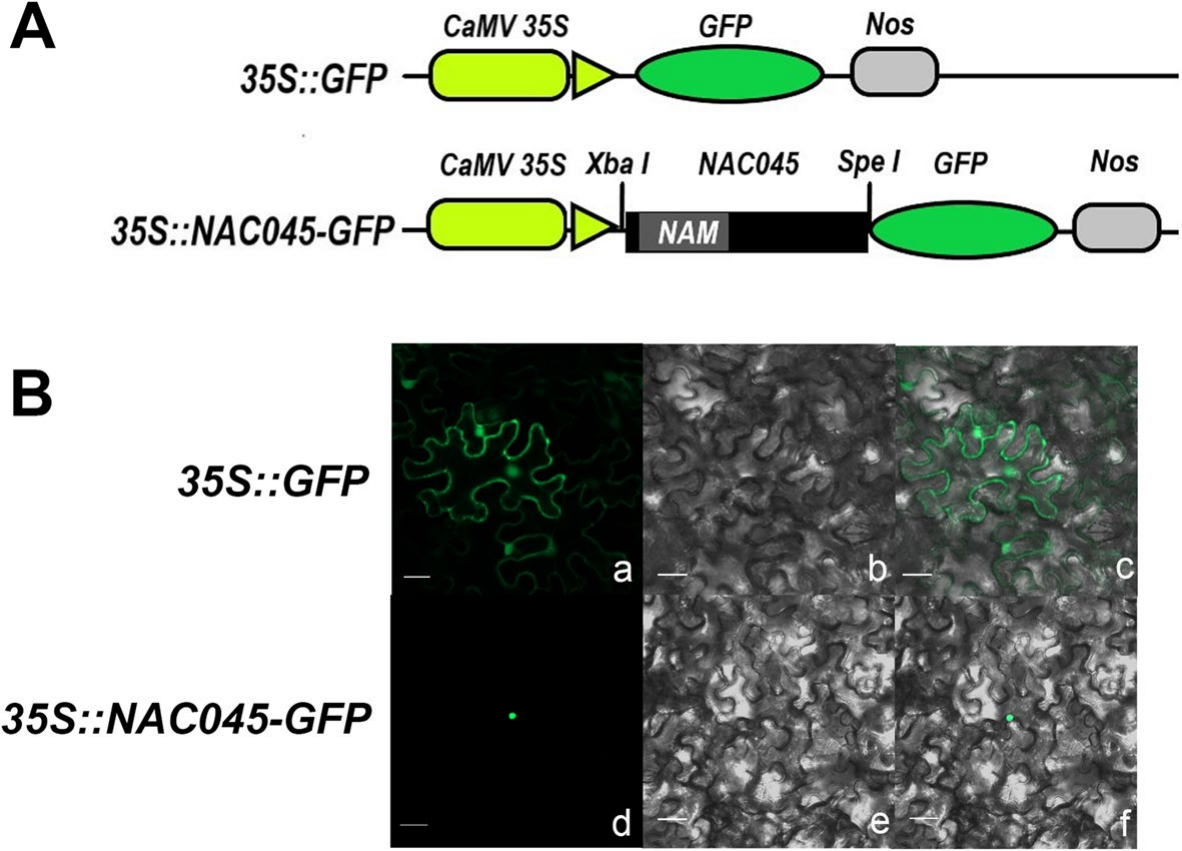
Subcellular localization of the *Pag*NAC045 protein. **A** Schematic map of the T-DNA inserted in the *35S::GFP* binary vector. **B** The *35S::NAC045-GFP* fusion construct and the positive control *35S::GFP* plasmid were introduced into tobacco epidermal cells. GFP fluorescence was observed by confocal laser scanning microscopy. (**a**) and (**d**) were fluorescence images observed in dark field (green), (**b**) and (**e**) were light images observed in bright field, (**c**) and (**f**) were merged images of dark field and bright field. Scale bar = 50 μm

According to the CELLO2GO prediction (Supplementary Fig. S1), the *Pag*NAC045 was localized in the nucleus. To confirm the result in vivo, *35S::NAC045-GFP* was constructed (Fig. 2A). The result of transient transformation of tobacco leaves showed that the fluorescence of NAC045-GFP was exclusively localized in the nucleus, while the positive control 35S::GFP was expressed throughout the whole cell (Fig. 2B). These results clearly showed that the *Pag*NAC045 was a nuclear-localized protein.

### Transactivation activity analysis of *Pag*NAC045 protein

**Fig. 3:**
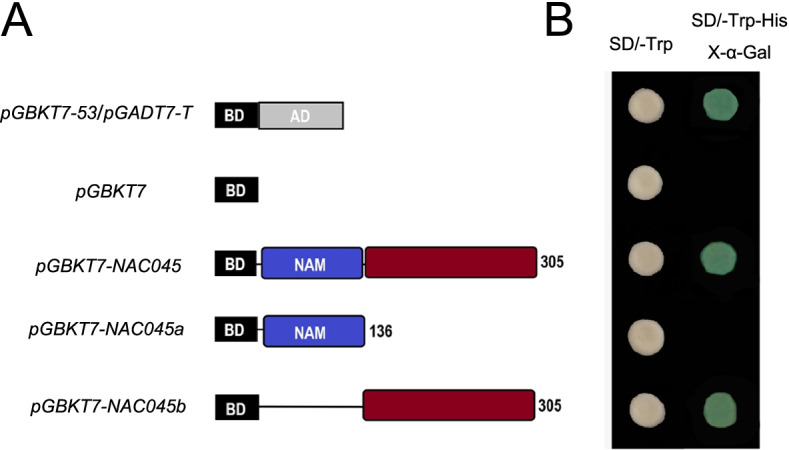
Transactivation analysis of the *Pag*NAC045 protein. **A** Schematic map of *pGBKT7-NAC045* (1-305 aa), *pGBKT7-NAC045a* (1-136 aa) and *pGBKT7-NAC045b* (137-305 aa) constructs. **B** Yeast colony assay showing transactivation activities of *pGBKT7-NAC045* (1-305 aa) and *pGBKT7-NAC045b* (137-305 aa)

To test the transactivation activity of poplar *Pag*NAC045 TF, the full length and truncated sequences of *PagNAC045* were cloned into *pGBKT7* vector, named *pGBKT7-NAC045* (1-305 aa), *pGBKT7-NAC045a* (1-136 aa) and *pGBKT7-NAC045b* (137-305 aa) (Fig. 3A). Transactivation assay showed that all the transformants including positive control *pGBKT7-53/pGADT7-T* and negative control *pGBKT7* can grow well on SD/−Trp selection medium, while only positive control, *pGBKT7-NAC045* and *pGBKT7-NAC045b* can grow and turn blue on SD/−Trp/−His/X-ɑ-Gal medium (Fig. 3B). The results showed that the *Pag*NAC045 had transactivation activity and the amino acid residues at the C-terminal part are essential for the activity.

### *PagNAC045* gene promoter analysis and its upstream regulator

**Fig. 4:**
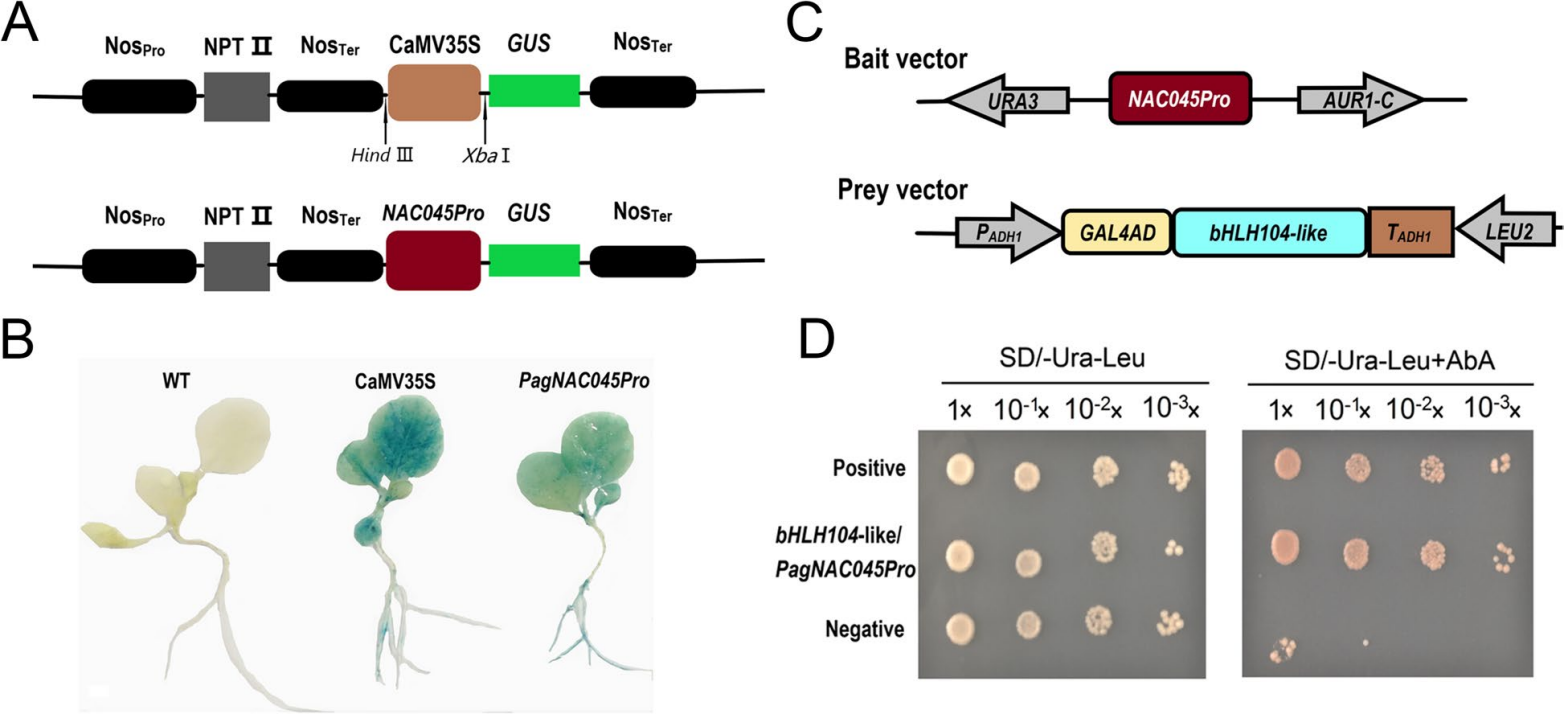
*PagNAC045* promoter analyses. **A** Schematic map of *PagNAC045* promoter that replaces the vector promoter CaMV35S; **B** *GUS* expression driven by the *PagNAC045* promoter in tobacco seedlings. **C** Schematic map of bait vector and prey vector. **D** Yeast one-hybrid assay to determine that the *PagNAC045* promoter was specially bound by a bHLH104-like transcription factor

To explore the function of the *PagNAC045* promoter, 1063 bp promoter sequence was ligated to the vector pBI121 (Fig. 4A) to drive the *GUS* gene expression, and the construct was used for tobacco transformation. *GUS* histochemical staining showed that the *PagNAC045 promoter* can drive *GUS* gene expression in the plant leaves and roots (Fig. 4B).

To discover the upstream regulators of *PagNAC045*, the promoter bait strain with *PagNAC045 promoter* was constructed (Fig. 4C) for cDNA library screening. A total of 48 positive colonies were selected by PCR with T7 primers (Supplementary Fig. S2). According to the blast sequence by NCBI Blast server, a TF gene *bHLH104*-like (XM_035069641.1) was selected. Based on the homolog sequence from *P. trichocarpa*, specific primers of *bHLH104*-like was designed (Supplementary Table S2) and the TF gene was isolated from the 84 K poplar. Then the full length of *bHLH104*-like gene was inserted into pGADT7 as a prey vector for further identification. The results of yeast-one hybrid assay revealed that the bHLH104-like protein can bind to the *PagNAC045* promoter sequence, i.e., it functions as an upstream regulator of the *PagNAC045* (Fig. 4D).

### Relative expression levels of *PagNAC045* in poplar

**Fig. 5:**
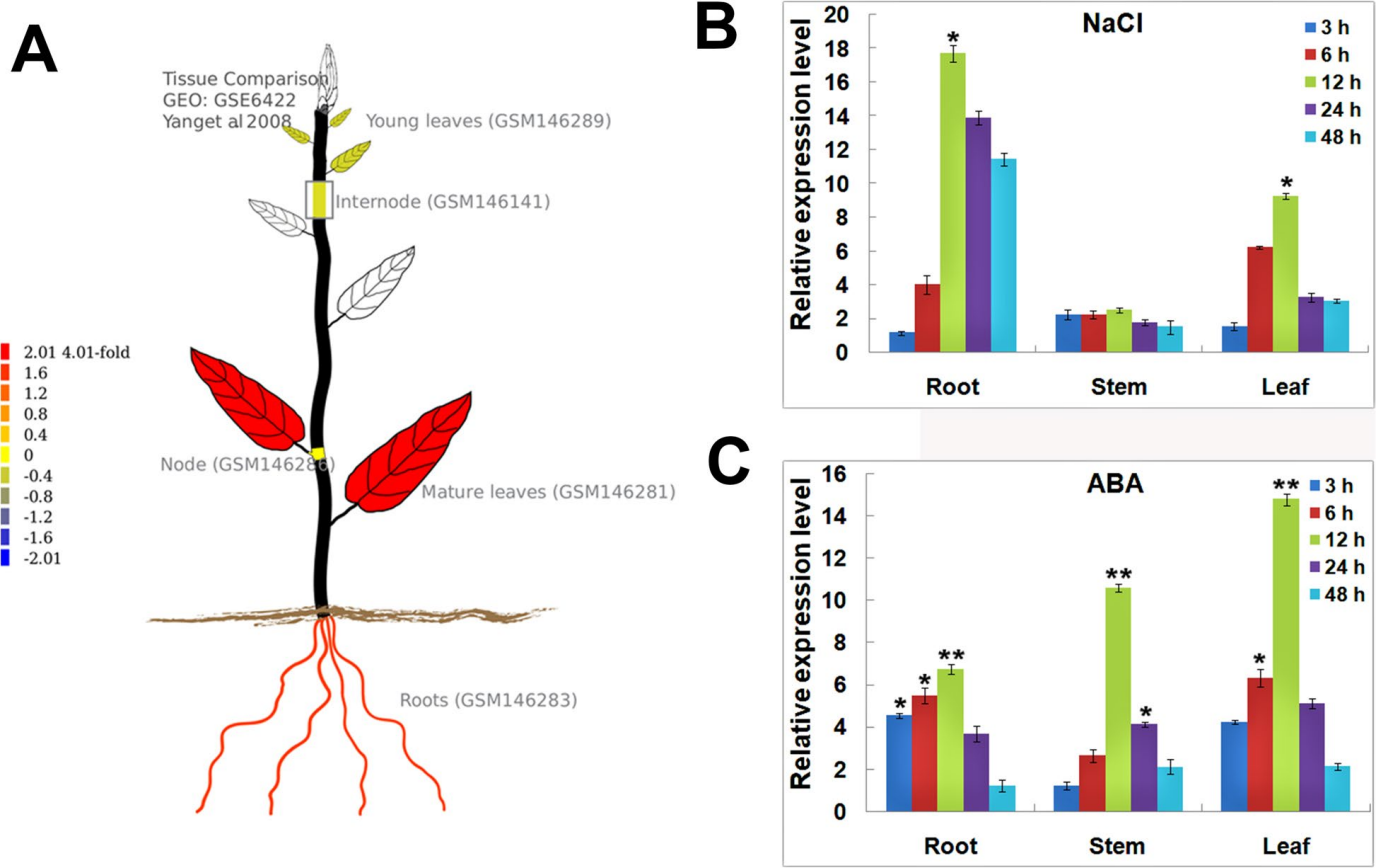
Tissue-specific expression pattern of *PagNAC045* in poplar. **A** Expression pattern of *PagNAC045* based on in silico prediction; **B** Relative expression levels of *PagNAC045* in root, stem, and leaf tissues under 150 mM NaCl treatment for 0, 3, 6, 12, 24 and 48 h; **C** after 50 μM ABA treatment for 3, 6,12,24 and 48 h. The 0 h time point was used as control. Student’s t-test: t: *, *P* < 0.05; **, *P* < 0.01. Error bars indicate mean ± SD

Based on in silico prediction, *PagNAC045* was highly expressed in mature leaves and roots, followed by internode and young leaves (Fig. 5A). To further analyze the relative expression levels of *PagNAC045* in different poplar tissues after salinity and ABA treatments, we collected roots, stems, and leaves under 150 mM NaCl or 50 μM ABA at 0, 3, 6, 12, 24 and 48 h, respectively. The results of RT-qPCR showed that the *PagNAC045* was significantly induced by salt and ABA in the roots and leaves of poplar, but not in stems when treated with salt. After treatment for 12 h, the expression level of *PagNAC045* reached the highest point, especially in roots under salt stress (Fig. 5B) and in leaves after ABA treatment (Fig. 5C).

### Identification of transgenic tobacco lines

**Fig. 6:**
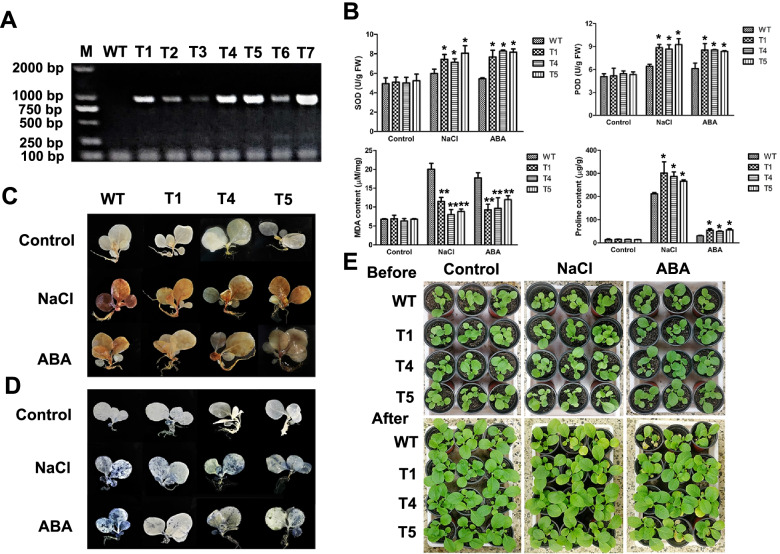
Identification and stress treatments of *PagNAC045* over-expressing transgenic tobacco lines. **A** RT-PCR validation of over-expressing transgenic lines with specific primers *PagNAC045F1* and *PagNAC045R1* (Supplementary Table S2); M, 2000 DNA marker; WT, wild type; T1-T6, transgenic lines; P, positive plasmid. **B** Biochemical analyses of superoxide dismutase (SOD), peroxidase (POD), malondialdehyde (MDA) and proline contents in the leaves of transgenic lines and WT. **C** Images of nitroblue tertazolium (NBT) staining. **D** Images of 3,3′-diaminobenzidine (DAB) staining. **E** Four-week-old tobacco plants in soil before the treatments (upper panels), and plants treated with 200 mM NaCl or 50 μM ABA for one week (bottom panels). Student’s t-test: t: *, *P* < 0.05; **, *P* < 0.01. Error bars indicate mean ± SD

Using an *Agrobacterium*-mediated leaf disc method, the transgenic plants with *PagNAC045-pBI121* recombinant construct were generated and selected on the selective 1/2 MS medium containing 100 mg/L kanamycin. According to PCR with the specific primers *PagNAC045F1* and *PagNAC045R1* (Supplementary Table S2) and semi-quantitative RT-PCR confirmation with *PagNAC045F4* and *PagNAC045R4*, six transgenic lines had the same length of band as that in the positive control, but the band was absent in the WT plants (Fig. 6A, Supplementary Fig. S3). In this study, transgenic lines T1, T4, and T5 were selected for further analysis.

### Salt and ABA tolerance of the transgenic tobacco plants

To investigate the functions of *PagNAC045*, three-week-old tobacco seedlings including WT and three transgenic lines T1, T4 and T5 were treated with 200 mM NaCl or 50 μM ABA for 12 h. Those treated with water were used as the control. The leaves were collected for SOD, POD, MDA, and proline measurements (Fig. 6B). The results showed that under control conditions, there were no significant changes of SOD, POD, MDA and proline contents between the WT and transgenic lines. However, after treatment with salt, the activities of SOD and POD, and proline content of the transgenic lines were 1.26 ± 0.06, 1.39 ± 0.04, and 1.35 ± 0.59 times of those of WT. In contrast, the MDA content of WT was 2.17 ± 0.31 times of the transgenic lines (Fig. 6B). Similarly, after ABA treatment, the activities of SOD and POD and proline content of transgenic lines were also increased to 1.48 ± 0.05, 1.38 ± 0.02 and 1.75 ± 0.76 times of those of WT. The MDA content of WT had a 1.74 ± 0.19 times increase compared to the transgenic lines (Fig. 6B).

Histochemical staining with DAB and NBT is often used to detect the accumulation of hydrogen peroxide (H_2_O_2_) and superoxide anion (O_2_^−^), respectively [41]. Three-week-old tobacco seedlings were irrigated with water, 200 mM NaCl or 50 μM ABA for 12 h. The whole seedlings were immersed in DAB and NBT staining solutions at 37 °C overnight. As shown in Fig. 6C and D, under control conditions, there was no significant difference between WT and transgenic lines, while after treatments with salt and ABA, WT showed more intense DAB- and NBT-stainings than the transgenic plants.

Based on the morphological phenotype, before the salt and ABA treatments the WT and transgenic lines grow indistinguishably. After treatments for 1 week, the transgenic lines grew obviously better than the WT (Fig. 6E).

### Expression of stress-related genes in the *PagNAC045* transgenic plants

**Fig. 7:**
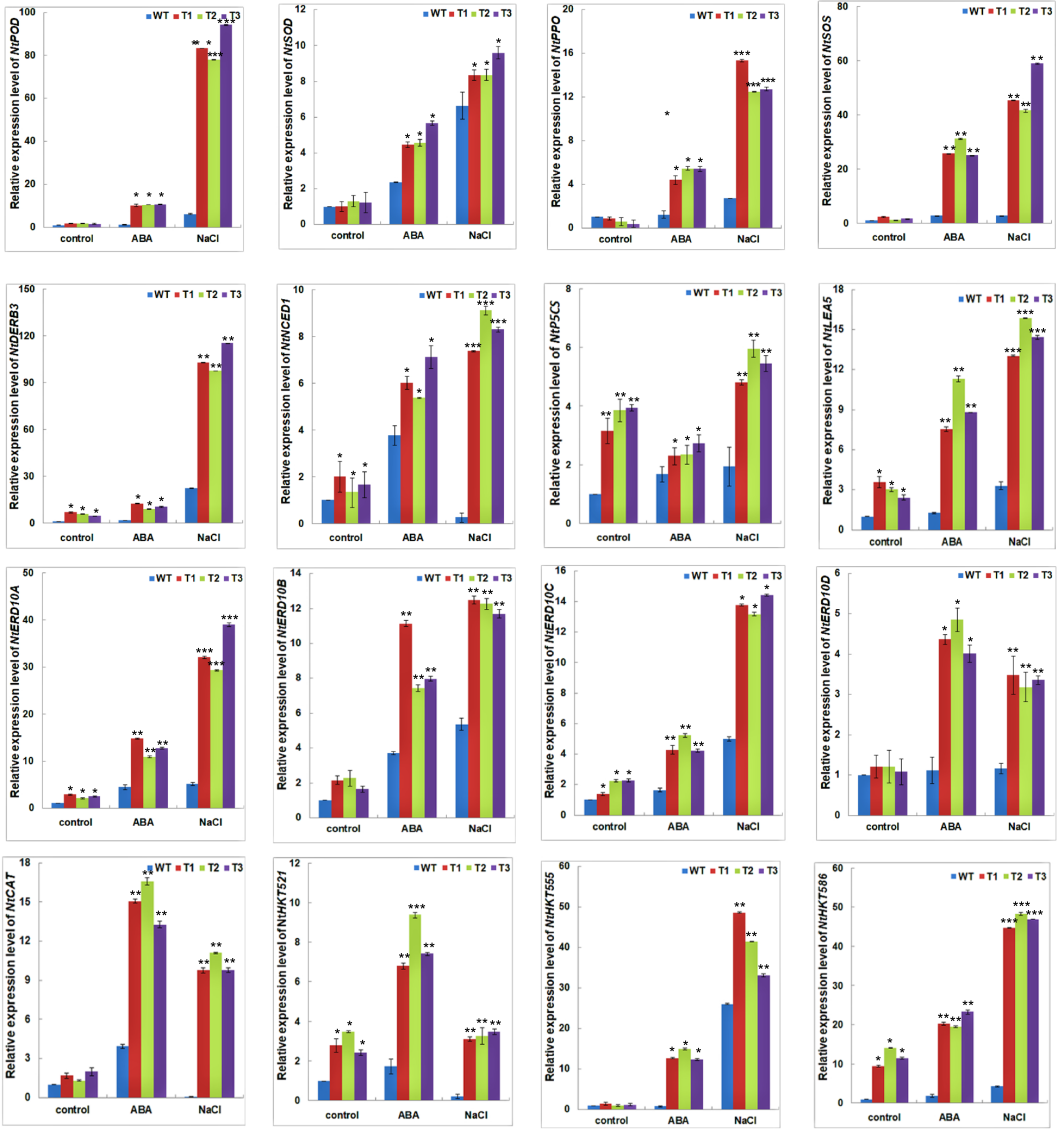
RT-qPCR-based gene expression profiling of stress-responsive genes under NaCl or ABA treatments of WT and *PagNAC045* transgenic seedlings. The 16 stress-related genes include *NtPOD*, *NtSOD*, *NtPPO*, *NtSOS*, *NtDREB3*, *NtNCED1*, *NtP5CS*, *NtLEA5*, *NtERD10A/B/C/D*, *NtCAT* and *NtHKT521/555/586*. Student’s t-test: t: *, *P* < 0.05; **, *P* < 0.01; ***, *P* < 0.001. Error bars indicate mean ± SD

The relative expression levels of 16 stress-related genes were analyzed after the tobacco seedlings were exposed to 50 μM ABA or 200 mM NaCl for 12 h. As shown in Fig. 7, under normal conditions, the expression levels of *NtPOD*, *NtSOD*, *NtPPO NtSOS* and *NtCAT* showed no differences between the WT and transgenic lines. However, under salt or ABA treatments, these five genes were significantly increased in transcripts. The upregulated expression levels of these genes under salt stress were much higher than those under ABA treatment. In addition, *NtDREB3*, *NtNCED1*, *NtP5CS*, *NtLEA5*, *NtERD10A/C* and *NtHKT521/586* were significantly upregulated in the transgenic lines compared to WT under normal conditions. Under salt stress, the relative expression levels of *NtDREB3*, *NtNCED1*, *NtP5CS* and *NtHKT521/555/586* were increased 105.48, 8.27, 5.42, 3.30, 41.10, and 46.75 folds, respectively, compared to WT, respectively. Under ABA treatment, the relative expression levels of these genes were increased 10.77, 6.18, 2.47, 7.87, 13.36 and 21.08 folds, respectively. Clearly, the stress-related genes displayed different expression patterns when responding to different stresses, and these differentially expressed genes may play vital roles in ABA and NaCl responses.

## Discussion

In poplar, 289 NAC genes were identified and can be clustered in to 15 distinct subgroups based on their deduced protein sequences [42]. Among these genes, 37 were significantly upregulated by salt stress according to the RNA-seq data [40]. Here we studied one of salt-induced NAC genes, *PagNAC045* from the 84 K poplar. It was expressed in different tissues of the poplar seedlings, and was significantly upregulated by NaCl and ABA in poplar roots and leaves, but not in stems. According to Yao et al [42], *PagNAC045* belongs to subgroup I of NAC TF family, and this gene has the highly conserved domain NAM at its N-terminus and transcriptional activation domain at its C-terminus. PPInetwork showed that *Pag*NAC045 may interact with eight candidate proteins and take a part in plant hormone signal transduction (Fig. 1). Four of them are related to ABA, including ABA-insensitive 5-like protein 7 (POPTR_0009s10400.1), ABA responsive elements-binding protein 2 (POPTR_0004s14790.1, ABF2-1), ABA-insensitive 5-like protein 5 isoform × 2 (POPTR_0014s02810.1) and bZIP transcription factor 6 family protein (POPTR_0002s12710.1). Thus, *Pag*NAC045 may be involved in ABA signaling pathway. The homologous gene of *PagNAC045* in *Arabidopsis* is *ATAF1* (AT1G01720.1). *ATAF1* was reported to be induced by drought, high salinity, ABA, methyl jasmonate, mechanical and wounding [43]. The results from this study showed that the expression level of *PagNAC045* in poplar was induced by salt and ABA, leading us to hypothesize the *PagNAC045* may have similar functions as *ATAF1* in *Arabidopsis* in the response of salt and ABA treatments. How *Pag*NAC045 is activated by salt and ABA, and what its targets are in the signaling pathways are not known.

It was reported that TFs as molecular switches can drive specific temporal and spatial gene expression to regulate diverse plant processes like stress signaling by binding to *cis*-acting elements in the promoter region [44, 45]. For the identification of upstream interacting factors, yeast one-hybrid was used to screen prey protein interactions according to the bait DNA promoter sequence [46]. For example, a potential TF which annotated as CRM-domain containing factor CFM3 and a sulfite oxidase-like protein in *Nicotiana tabacum* were identified to bind to the promoter of nitrate reductase gene [47]. A VASCULAR-RELATED NAC DOMAIN7 can bind to HD-ZIPIII TFs *REV* and *PHB* promoters in *Arabidopsis* [48]. The bZIP TF TGA9 can upregulate the expression of autophagy-related *ATG8B* and *ATG8E* by binding the special sites TGA in their promoters [49]. In our study, among the 48 positive colonies selected, a basis helix-loop-helix (bHLH) 104 TF was found to act as a positive regulator in ABA signaling pathway [50] and confers response to salinity stress [51]. Our results showed that bHLH104 TF can bind to the upstream of *PagNAC045* that would function as a positive regulator to activate the expression of *PagNAC045*, especially under stress treatment.

Most stress adaptive mechanisms in plants are accompanied by certain morphological and physiological changes [52], especially, the ROS accumulation in various forms like hydrogen peroxide (H_2_O_2_), superoxide anions, hydroxyl radical (OH^−^) and singlet oxygen (^1^O^2^) [53]. SOD and POD are key antioxidant enzymes for ROS-scavenging and protecting different cellular structures from damage under stress conditions [54]. MDA is commonly used to determine the level of oxidative stress in plants [55]. NBT and DAB are used for measuring superoxide anion and H_2_O_2_, respectively [56]. Free proline is another useful index to monitor plant physiological status and evaluate the stress tolerance [57]. In this study, tobacco seedlings with *PagNAC045* overexpression displayed higher activities of POD and SOD, and higher proline content than WT when challenged with NaCl and ABA. Together with the lower MDA and ROS levels in the transgenic plants than WT, these results indicate that *PagNAC045* plays a positive role in salt and ABA response through activating cellular antioxidant system to scavenge excess ROS.

**Fig. 8:**
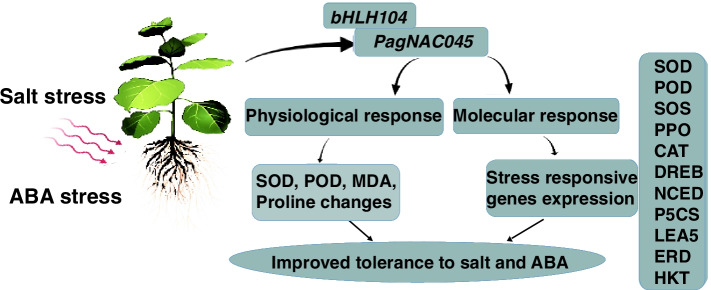
Schematic presentation of *PagNAC045* in plant salt and ABA tolerance. SOD, superoxide dismutase; POD, peroxidase; MDA, malondialdehyde; SOS, plasmalemma Na^+^/H^+^antiporter; PPO, polyphenol oxidase; CAT, ascorbate peroxidase; DREB, dehydration responsive element binding protein; NCED, 9-cis-epoxycarotenoid dioxygenase1; P5CS, 1-pyrroline-5-carhoxylate synthetase; LEA5, late-embryogenesis-abundant protein5; ERD, early responsive to dehydration; HKT, high-affinity potassium transporter

Salt stress can alter the expression of many stress-responsive genes [23]. It was reported that salt stress induced 932 genes and repressed 367 genes in *Arabidopsis* based on transcriptomics [57]. Similarity, ABA activated the expression of many stress-related genes in plants [58]. Here we selected 16 stress-related genes and quantified their relative expression levels under salt or ABA treatment. Among them, two ROS scavenging *NtSOD* and *NtPOD* expression levels were significantly increased under salt and ABA treatments. The results were consistent with the transcriptional regulation of the enzymatic activities (Fig. 6B). Polyphenol oxidase (PPO) is a ubiquitous enzyme for catalyzing the oxidation of phenols to highly reactive quinones. It has been confirmed to be induced by wounding, ABA, and methyl jasmonate (MeJA) in tobacco plants [59]. In the *PagNAC045* overexpressing transgenic tobacco, *NtPPO* was significantly induced by ABA and salt stress. This is a strong indication that *Pag*NAC045 could regulate the expression of *NtPPO* to decrease the stress damage. The salt overly sensitive (SOS) pathway is the key pathway for regulating Na^+^/K^+^ ion homeostasis to decrease ionic damage in response to salt stress [60]. In the transgenic lines, no difference in *NtSOS* expression was observed under normal conditions. However, after treatment with salt and ABA, *NtSOS* expression was significantly increased (Fig. 7). This result suggests that *Pag*NAC045 may function to activate *NtSOS* under stress conditions. *NtDREB3* is C-repeat-binding-factor/dehydration-responsive element (CBF/DRE) TF, *NtERD10A/B* are downstream CBF/DRE-binding regulon, and *NtERD10C/D* are early dehydration-responsive genes [61]. The levels of these transcripts were significantly lower in WT than those in the *PagNAC045* transgenic plants, indicating these genes may be targets of the *Pag*NAC045 TF. *NtP5CS* encodes an enzyme to catalyze the first two steps in proline biosynthesis [62]. The elevated contents of proline in the transgenic lines under salt and ABA treatments may be attributed to the upregulation of *NtP5CS* in the transgenic tobacco (Fig. 7). *NtLEA5* gene encodes a group 5 late embryogenesis abundant (LEA) protein, which functions in stabilizing labile enzymes and protects the structure of macromolecule and membranes [63]. The high affinity K^+^ transporter (HKT) works in limiting Na^+^ accumulation and minimizing the osmotic imbalance [64]. It was demonstrated that HKT can improve the salt tolerance in Populus [65]. All these genes were significantly induced by salt and ABA in the transgenic tobacco lines. These results suggest that overexpression of *PagNAC045* facilitates the tolerance to salt and ABA by regulating the expression of downstream stress-related genes. Further analyses are needed to elucidate the molecular actions of *PagNAC045* in regulating downstream genes when plants experience abiotic stresses (Fig. 8).

## Conclusions

In this study, we isolated the *PagNAC045* gene from 84 K poplar, which was induced by salt stress and ABA treatment. The characteristics of its protein sequence and structure were analyzed. The *Pag*NAC045 was a nucleus-localized protein and had transactivation domain in its C-terminus. In addition, the promoter of *PagNAC045* can drive *GUS* gene expression and a bHLH104 TF was upstream of *PagNAC045* function. Furthermore, the *PagNAC045* overexpressing tobacco plants were generated. Biochemical analyses of ROS antioxidant enzymes SOD and POD, and the contents of proline and MDA of the transgenic tobacco seedlings showed that overexpression of *PagNAC045* significantly increased the ROS scavenging ability and thereby reduced cellular membrane damage. Moreover, the *PagNAC045* differentially regulated the expression of stress-related genes in the transgenic tobacco plants when treated with NaCl and ABA. Our results provide important insight into the molecular mechanisms underlying the *Pag*NAC045 functions in plant response to salt and ABA (Fig. 8).

## Materials and methods

### Plant materials

*P. alba×P. glandulosa* (84 K poplar) seedlings were cultured on 1/2 MS (Murashige and Skoog medium) plant medium (pH 5.7) supplemented with 0.1 mg/mL indole-3-butytric acid (IBA) and 0.01 mg/mL 1-naphthaleneacetic acid (NAA) and placed in a growth chamber with a photoperiod of 16/8-h light/dark cycle and an average temperature of 25 °C [35]. For tobacco transformation, *Nicotiana benthamiana* was used. The seeds were sterilized with 20% bleach for 15 min and washed with sterile water for 5 times, and then grown on the MS medium plates for 7 days under the growth chamber conditions. The seedlings with four true leaves were transferred to transparent glass bottle with 100 mL MS medium for a month. The fully expanded leaves were cut into 1 × 1 cm squares for leaf dish transformation. The materials poplar and tobacco used in this study were planted in the experimental field of Northeast Forestry University, Harbin, China.

### Cloning and sequence analysis of *PagNAC045*

RNA was extracted from the 84 K poplar leaves and then reverse transcripted into cDNA following instructions included in a Prime Script RT reagent kit (Takara, China). According to the sequence of a highly homologous gene *NAC045* (Potri.007G099400.1) of *Populus trichocarpa* from the Plant Transcription Factor Database v5.0 (http://planttfdb.gao-lab.org/), specific primes *PagNAC045F1* and *PagNAC045R1* (Supplementary Table S2) were designed to amplify the gene *PagNAC045* from cDNA of 84 K poplar by polymerase chain reaction (PCR). Based on the translated amino acid sequence, the conserved domain and motifs of *Pag*NAC045 were analyzed using NCBI conserved domain search (https://www.ncbi.nlm.nih.gov/Structure/cdd/wrpsb.cgi) and MEME Suite 5.3.0 (http://meme-suite.org/index.html). *Pag*NAC045 protein structure was predicted by SWISS-MODEL (https://swissmodel.expasy.org/interactive/NHZXxy/models/). Also, we found some homologous proteins by NCBI blast and constructed a phylogenetic tree using MEGA7.0 with a Neighbor-Joining method. In addition, through STRING database, we analyzed the potential interactions of proteins, and constructed the protein-protein interactions (PPI) network.

### Subcellular localization analysis of *Pag*NAC045 protein

CELLO2GO (http://cello.life.nctu.edu.tw/cello2go/) for protein subcellular localization prediction with ontology annotation was used for the subcellular localization analysis. To experimentally test the result from CELLO2GO, the full length of *PagNAC045* sequence without terminal codon was amplified with primers *PagNAC045F2* and *PagNAC045R2* including restriction enzyme sites of *Xba I* (TCTAGA) and *Spe I* (ACTAGT), respectively. After ligation to the vector pBI121 with green florescence protein (GFP), a construct *35S::NAC045-GFP* was produced. The plasmid of *35S::NAC045-GFP* was transformed into agrobacteria strain GV3101 for tobacco infiltration. The plasmid of *35S::GFP* was used as positive control. Two days later, the GFP signal was observed by confocal laser scanning microscope (LSM 700, Zeiss, Germany).

### Transcriptional activation assay

Some of NAC family proteins are plant-specific transcription factors [66]. To test whether *Pag*NAC045 protein has transcriptional activation activity and the activation domain, the full length of *Pag*NAC045 (0-305 aa), the highly conserved domain NAM (0-136 aa), the sequence at the carboxy-terminal domain (137-305 aa) were ligated to *GAL4 DNA-BD* vector *pGBKT7*, called *pGBKT7-NAC045*, *pGBKT7-NAC045a* and *pGBKT7-NAC045b*, respectively. All the primers for gene amplification were listed in Supplementary Table S2. These fusion plasmid vectors with different fragments were transformed into Y2H yeast strain and screened on selective medium without *Trp* and *His* plates. *β*-Galactosidase assays were then performed on filter lifts of the colonies to detect activation of the *lacZ* reporter gene. *pGBKT7* and *pGBKT7-53/pGBKT7-T* were used as negative and positive controls, respectively.

### *PagNAC045* promoter analysis and yeast-one hybrid

*Cis*-acting elements in promoters function as binding sites of TFs and are important for transcription initiation [67]. To isolate and explore the function of *PagNAC045* promoter (*PagNAC045Pro*), we extracted DNA from the 84 K poplar using a NuClean PlantGen DNA Kit (CWBIO, China). Specific primers for promoter cloning were designed as follows, *PagNAC045F3* and *PagNAC045R3* with restriction enzyme sites *Cla I* and *Xba I*, respectively (Supplementary Table S2). After PCR amplification, the 1063 bp *PagNAC045* promoter sequence was ligated to a plant binary expression vector pBI121 with CaMV35S to drive β-glucuronidase (*GUS*) gene expression (Fig. 4A). The recombinant plasmid *PagNAC045Pro* and positive control CaMV35S were transformed into agrobacteria EHA105 strain and used for tobacco transient transformation. The agrobacteria were cultured in LB liquid medium which contains 50 mg/L rifampin and 50 mg/L kanamycin overnight. The agrobacteria were harvested by centrifugation for 10 min at the speed of 5000 rpm, resuspended in 1/2 liquid MS containing 150 μM acetosyringone until OD_600_ reached 0.2. Two-week-old tobacco seedlings were immersed in the agrobacteria suspension for 2 days at 25 °C. Then the seedlings were transferred to a X-Gluc staining solution (50 mM NaH_2_PO_4_, 50 mM Na_2_HPO_4_, 10 mM Na_2_EDTA, 0.1% TritonX-100, 10 mM K_3_[Fe (CN)_6_], 10 mM K_4_[Fe (CN)_6_] and 20 mM X-Gluc) and shaked at the speed of 120 rpm at 37 °C overnight. After washing the seedlings with a destaining solution (ethanol/acetic acid, V/V = 3:1) for three times, the blue coloring reflecting the *GUS* activity could be observed.

To further explore the interactions between *PagNAC045* promoter and its TF in poplar, yeast one-hybrid assay was performed to map TF-DNA interactions [68] based on the manufacturer’s instructions of Matchmaker Gold Yeast one-hybrid Library Screening System (Clontech, USA) and Yao et al. [69]. The 1063 bp *PagNAC045Pro* sequence was inserted into pAbAi bait vector, and the recombinant bait vector was then transformed into Y1HGold yeast strain and selected on synthetic dextrose (SD)/−Ura solid culture medium. A poplar cDNA library was constructed as the method of Matchmaker™ Gold Yeast One-Hybrid Library Screening System (Clontech, USA). The double-stranded cDNA which was synthesized through long-distance (LD)-PCR and *Sma I*-linearized vector pGADT7-Rec was co-transformed into Y1HGold with bait *PagNAC045Pro* and selected on SD/−Leu/AbA solid medium at 30 °C for 3-5 days. Well-grown single colonies were cultured and identified by PCR with *T7* primers (Supplementary Table S2). The PCR products were sequenced, and bioinformatics analyses were used for identification of positive clones. To further confirm the interaction between *PagNAC045Pro* and its potential TF, primers were designed and used for amplification of target TF gene from poplar based on the blasted sequence. The full length of target TF sequence was inserted into pGADT7 as the prey vector (Fig. 4C) and co-transformed into Y1HGold yeast strain with the bait vector *PagNAC045Pro-pAbAi*. The interaction between *PagNAC045Pro* and its TF was determined based on the yeast growth on the selective media.

### Spatial and temporal quantitative expression analysis of *PagNAC045*

To analyze the expression levels of *PagNAC045* in different tissues, we predicted the expression patterns in different tissues of *PagNAC045* in silico using an exlmage tool of the PopGenIE V3 database (https://popgenie.org/gene?id=Potri.007G099400). To test the results, one-month-old poplar seedlings from culture medium were taken out and watered with 150 mM NaCl or 50 μM ABA for 0, 3, 6, 12, 24 and 48 h. Root, stem and leaf tissues were harvested with three biological replicates at each time point of control and the two treatments for RNA extraction and RT-qPCR. The *Actin* was used as the reference gene in RT-qPCR and the primers *AF* and *AR* were shown in Supplementary Table S2. The relative expression level in different samples was calculated using a 2^−ΔΔCt^ method [70].

### Generation of transgenic tobacco lines

One-month-old WT tobacco seedlings in culture medium were used for leaf discs transformation by agrobacteria-mediated transgenic approach [71, 72]. The full length of *PagNAC045* sequence was cloned and ligated to plant expression vector pBI121 and transformed into agrobacteria strain EH105. The transformed agrobacteria were cultured in LB liquid medium containing 50 mg/L rifampin and 50 mg/L kanamycin overnight until OD_600_ = 0.6. The tobacco leaves were immersed in bacteria solution for 15 min and then screened on MS medium with 100 mg/L kanamycin. To identify the transgenic tobacco plants, the leaves from different lines were collected for RNA extraction and PCR detection. WT was used as a negative control. To further confirm the transgenic tobacco lines, semi-quantitative assay was conducted, the *Ntactin* was the reference primers for RT-PCR. The specific primers *PagNAC045F4* and *PagNAC045R4* (Supplementary Table S2) were designed, and cDNAs of each line were collected as template for semi-quantitative RT-PCR. The identified seedlings were transplanted into soil under greenhouse conditions for seeds and the third-generation seeds were used for further experiment.

### Salt and ABA treatment of transgenic tobacco plants

One-month-old tobacco plants in soil were irrigated with 200 mM NaCl or 50 μM ABA treatments for 1 week. Three transgenic lines (T1, T4 and T5) and WT were used. The leaves from different lines under control, salt and ABA stress were collected for physiological measurements. The physiological indexes including superoxide dismutase (SOD), peroxidase (POD), malondialdehyde (MDA) and proline contents were measured according to published methods [35]. Histochemical analyses including nitroblue tertazolium (NBT) staining and 3,3′-Diaminobenzidine (DAB) staining were conducted according to the methods in Sekulska et al. [73].

### Stress-related genes analysis in transgenic plants

The expression levels of stress-related genes including peroxidase *NtPOD*, superoxide dismutase *NtSOD*, polyphenol oxidase *NtPPO*, plasmalemma Na^+^/H^+^antiporter *NtSOS*, regulatory proteins *NtDREB3*, 9-cis-epoxycarotenoid dioxygenase1 *NtNCED1*, 1-pyrroline-5-carhoxylate synthetase *NtP5CS*, late-embryogenesis-abundant protein5 *NtLEA5*, early responsive to dehydration *NtERD10A/B/C/D*, ascorbate peroxidase *NtCAT* and Na^+^ antiporter genes *NtHKT521/555/586* were profiled using RT-qPCR quantification. *Ntactin* and *NtUbiquitin* were used as reference genes. Leaves were harvested with three biological replicates for each sample. The related primer sequences were listed in Supplementary Table S3. The relative expression level in different samples was profiled and calculated by a 2^−ΔΔCt^ method as previously described [69].

## Supplementary Information

**Additional file 1.**

## Data Availability

The datasets generated and /or analyzed during the current study are available in NCBI SRA with the accession number SRP267437.

## Abbreviations

84 K: *Populus alba×P. glandulosa*
TFs: Transcription factors
MS: Murashige and Skoog medium
IBA: Indole-3-butytric acid
NAA: 1-naphthaleneacetic acid
PCR: Polymerase chain reaction
PPI: Protein-protein interactions
GFP: Green florescence protein
*GUS*: β-glucuronidase
SOD: Superoxide dismutase
POD: Peroxidase
MDA: Malondialdehyde
NBT: Nitroblue tetrazolium
DAB: 3,3′-Diaminobenzidine
