# Supplementary material for: Functional analysis of PagNAC045 transcription factor that improves salt and ABA tolerance in transgenic tobacco
Source: BMC Plant Biol. 2022 May 25;22:261. doi: 10.1186/s12870-022-03623-8 (PMC9131654; doi:10.1186/s12870-022-03623-8)
Supplement: Supplementary file 1 — Additional file 1. [file 12870_2022_3623_MOESM1_ESM.docx]

**Supplementary files**

**
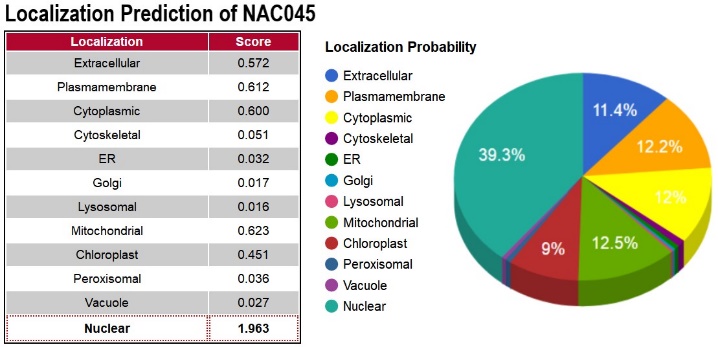
**

**Supplementary Figure S1** Localization prediction of *Pag*NAC045 by CELLO2GO

**
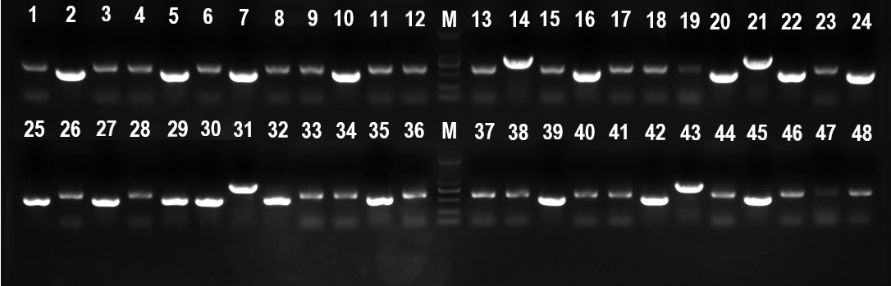
**

**Supplementary Figure S2** PCR identification of positive colonies from yeast one-hybrid assay

**
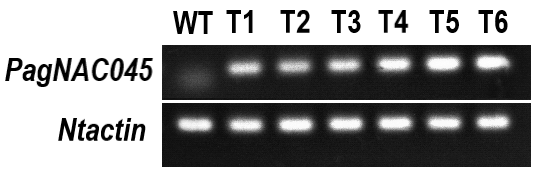
**

**Supplementary Figure S3 Semi-quantitative identification of transgenic tobacco lines. WT: wild-type; T1-6: transgenic tobacco lines; actin: reference gene in tobacco.**

**Supplementary Table S1** Predicted partners of PPI

| **Predicted partners** | **Annotated Keywords** | **Score** |
| --- | --- | --- |
| POPTR_0002s19420.1 | Transcription factor upbeat1 | 0.669 |
| POPTR_0014s11360.1 | Uncharacterized protein loc7495208 | 0.669 |
| POPTR_0010s15750.1 | Probable WRKY transcription factor 48 | 0.620 |
| POPTR_0001s06170.1 | GLUCOSE INSENSITIVE 1 family protein | 0.619 |
| POPTR_0016s10120.1 | Tau class glutathione transferase GSTU45 | 0.617 |
| POPTR_0010s20570.1 | DC1 domain-containing family protein; Cysteine/Histidine-rich C1 domain family protein | 0.611 |
| POPTR_0004s00890.1 | Probable WRKY transcription factor 31; WRKY transcription factor 6 family protein | 0.604 |
| POPTR_0006s13530.1 | Protein reveille 8 isoform x3 | 0.604 |
| POPTR_0008s10280.1 | Probable WRKY transcription factor 48 isoform x1 | 0.604 |
| POPTR_0016s08470.1 | Protein reveille 8 isoform x1 | 0.604 |
| POPTR_0001s27370.1 | Zinc finger ccch domain-containing protein 30 | 0.602 |
| POPTR_0009s06580.1 | Zinc finger ccch domain-containing protein 30 | 0.602 |
| POPTR_0002s12710.1 | BZIP transcription factor 6 family protein | 0.600 |
| POPTR_0004s08900.1 | Uncharacterized protein | 0.600 |
| ABF2-1 | Abscisic acid-insensitive 5-like protein 7 isoform x1; Abscisic acid responsive elements-binding protein 2 | 0.600 |
| POPTR_0004s20760.1 | Glutathione s-transferase u9; Glutathione S-transferase GST 12 family protein | 0.600 |
| POPTR_0005s14550.1 | Glutathione s-transferase dhar3, chloroplastic; Belongs to the GST superfamily | 0.600 |
| SMH905 | Telomere repeat-binding factor 2 isoform x3; Single myb histone | 0.600 |
| POPTR_0008s13470.1 | Glutathione s-transferase u9 | 0.600 |
| POPTR_0009s10400.1 | Low quality protein: abscisic acid-insensitive 5-like protein 7 | 0.600 |
| MYB180 | Transcription factor myb, plant | 0.600 |
| SMH906 | Telomere repeat-binding factor 2 isoform x3; Single myb histone | 0.600 |
| POPTR_0014s02810.1 | Abscisic acid-insensitive 5-like protein 5 isoform x2; BZIP transcription factor 6 family protein | 0.600 |
| MYB168 | Myb family transcription factor family protein | 0.600 |
| POPTR_0001s13240.1 | Jasmonate zim domain-containing protein | 0.599 |
| POPTR_0002s08920.1 | Probable calcium-binding protein cml29 | 0.599 |
| POPTR_0003s16350.1 | Jasmonate zim domain-containing protein | 0.599 |
| POPTR_0017s02090.1 | Putative glutathione S-transferase family protein; Belongs to the GST superfamily | 0.599 |
| POPTR_0006s24330.1 | Heat shock transcription factor, other eukaryote | 0.598 |
| POPTR_0001s22660.1 | Transcription factor srm1 | 0.581 |
| POPTR_0008s01730.1 | Syringolide-induced protein 1-3-1B | 0.581 |
| POPTR_0010s24710.1 | Transcription factor srm1 | 0.581 |
| POPTR_0003s16300.1 | Transcription factor bHLH137 | 0.570 |
| POPTR_0012s10670.1 | Basic helix-loop-helix family protein | 0.570 |
| POPTR_0015s11550.1 | Transcription factor bHLH137 isoform x3; Basic helix-loop-helix family protein | 0.570 |
| POPTR_0021s00280.1 | Probable WRKY transcription factor 31 isoform x3; WRKY transcription factor 42 family protein | 0.560 |
| GH3-12 | Jasmonic acid-amido synthetase jar1 | 0.559 |
| GH3-13 | Jasmonic acid-amido synthetase jar1; GH3 family protein | 0.559 |
| POPTR_0001s02140.1 | annotation not available | 0.551 |
| POPTR_0003s09370.1 | Heat shock transcription factor, other eukaryote; Heat shock transcription factor family protein | 0.551 |

**Supplementary Table S2** Primers information for vectors construction

| **Primer name** | **Seuquence (5’-3’)** | **Purpose** |
| --- | --- | --- |
| *PagNAC045F1* | GCAGCAGCAGCAAGAACAAGCAGC | *PagNAC045* cloning from 84K poplar |
| *PagNAC045R1* | GCCACTGGGCACACATCAAGAACC |  |
| *PagNAC045F2* | GCGTCTAGAGCAGCAGCAGCAGCAAGAACAAGC | Recombinant vector *35S::NAC045-GFP* construction, the underlined sequence *Xba I* and *Spe I* sites, respectively |
| *PagNAC045R2* | GCGACTAGT GGCTTCTGTAAATACATGAAC |  |
| *pGBKT7-NAC045F* | GAATTCATGAAAGGAAATAGATCAGCAG | construction of recombinant vector pGBKT7-NAC045, the underlines were restriction enzyme sites *EcoR I* and *Sal I*, respectively |
| *pGBKT7-NAC045R* | GTCGACTCAAAATGGCTTCTGTAAATAC |  |
| *pGBKT7-NAC045aF* | GAATTCATGAAAGGAAATAGATCAGCAG | construction of recombinant vector pGBKT7-NAC045a, the underlines were restriction enzyme sites *EcoR I* and *Sal I*, respectively |
| *pGBKT7-NAC045aR* | GTCGACCGGCAGTCCTGTCAACATTAGC |  |
| *pGBKT7-NAC045bF* | GAATTCGCTAATGTTGACAGGACTGCCG | construction of recombinant vector pGBKT7-NAC045a, the underlines were restriction enzyme sites *EcoR I* and *Sal I*, respectively |
| *pGBKT7-NAC045bR* | GTCGACTCAAAATGGCTTCTGTAAATAC |  |
| *PagNAC045F3* | GCGATCGATGGTGGCGACGACTCCTGGAGCCCG GTCCACGACATCCAAAACCC | construction of *PagNAC045* overexpression vector, the underlines were restriction enzyme sites *Cla Ⅰ* and *Xba Ⅰ*, respectively |
| *PagNAC045R3* | GCGTCTAGACATTACCAGTTGGTCTGGTGTCAA CGTTCTTGGTGCTTTTCTTC |  |
| *T7F* | AATACGACTCACTATAGGGCG | identification of positive colonies in yeast one-hybrid assay |
| *T7R* | AGATGGTGCACGATGCACAG |  |
| *bHLH104F* | GAATTCATGGAAACAATAGATGAGATTGAG | *bHLH104*-like gene cloning from 84K poplar and then insert into the yeast vector pGADT7, the underlines were restriction enzyme sites *EcoR I* and *BamH I*, respectively. |
| *bHLH104R* | GGATCCTGCAGCAGGTGGCCTGAGCTCATG |  |
| *AF* | ACCCTCCAATCCAGACACTG | The primers of reference gene *Actin* for poplar RT-qPCR measurement |
| *AR* | TTGCTGACCGTATGAGCAAG |  |
| *PagNAC045F4* | CTGGTAAAGCTCCTAAAG | The primers for semi-quantitative identification |
| *PagNAC045F4* | CATCTCTGGATACTTTGC |  |

**Supplementary Table S3** Primers information of stressed-related genes

| **Primer name** | **Accession number** | **Forward (5’-3’)** | **Reversed (5’-3’)** |
| --- | --- | --- | --- |
| *Ntactin* | U60489 | CATTGGCGCTGAGAGATTC | GCAGCTTCCATTCCGATCA |
| *NtUbiquitin* | U66264.1 | AAAGAGTCAACCCGTCACCT | ACATCACGACCACAACCAGA |
| *NtPOD* | AB178953 | CTCCATTTCCATGACTGCTTTG | GTTGGGTGGTGAGGTCTTT |
| *NtSOD* | AB093097 | CGGCAATTAGCGGTGACATA | ATGGCGTCATGTAGCTGTTC |
| *NtPPO* | A27686.1 | AACCCGTTCCGTGTGAAAGTCC | CTTCGATTACGCACCGATGCCA |
| *NtSOS* | LOC107768444 | TCCCAAAGAATAGGTGCC | TGGATGACGAAGAACCACT |
| *NtNCED1* | HM068892 | ACGAACTCCAACACCCTTTAC | AGGGAGTGAGAGACTGGATTT |
| *NtP5CS* | HM854026 | GACACGGACTGATGGAAGATTAG | GCACCTGAAGTCACCAGAATAA |
| *NtDERB3* | EU727157 | GCCGGAATACACAGGAGAAG | CCAATTTGGGAACACTGAGG |
| *NtLEA5* | AF053076 | GTTACCATACCACGTCCCATAG | GAGCTAGGACGCTCCATATTT |
| NtERD10A | AB049335 | TCTGAAGCGTGGCACTATTT | TCCACGGCACATCACTATAAC |
| NtERD10B | AB049336 | CAACTGCAACAACTACGACT | GGTGGCCAGGAAGCTTCT |
| *NtERD10C* | AB049337 | AACGTGGAGGCTACAGATCG | GTTCCTCTTGGGCATGAGTT |
| *NtERD10D* | AB049338 | GAGGACACGGCTGTACCAGT | GCGCCACTTCCTCTGTCTT |
| *NtCAT* | NM_001325673 | AGGTACCGCTCATTCACACC | AAGCAAGCTTTTGACCCAGA |
| *NtHKT521* | LOC107783521 | ATCATCTTACCGCCAAGG | GGCAGCAGAAACAGAAGTG |
| *NtHKT555* | LOC107787555 | AACCTCCACCTTCGCTATT | GAACCCAAACACCGTAACC |
| *NtHKT586* | LOC107781586 | GCCTCCACAAATCCATTC | TGCTTGAGACAGTTACCGAA |

*Ntactin* and *NtUbiquitin* were reference genes for RT-qPCR.
